# Effect of Combined Soil Amendment on Immobilization of Bioavailable As and Pb in Paddy Soil

**DOI:** 10.3390/toxics10020090

**Published:** 2022-02-16

**Authors:** Young-Kyu Hong, Jin-Wook Kim, Sang-Phil Lee, Jae-E. Yang, Sung-Chul Kim

**Affiliations:** 1 Department of Bio-Environmental Chemistry, Chungnam National University, Daejeon 34134, Korea; hyk102030@naver.com (Y.-K.H.); kin1888@naver.com (J.-W.K.); 2 Department of Biological Environment, Kangwon National University, Chuncheon 24341, Korea; tlsehd77@kangwon.ac.kr

**Keywords:** bioavailability, heavy metal pollution, correlation analysis, soil amendments

## Abstract

Heavy metal pollution in soil can have detrimental effects on soil ecosystems and human health. In situ remediation techniques are widely used to reduce the bioavailable fractions of heavy metals in soil. The main objective of this study was to examine the reduction of the bioavailable fractions of As and Pb in paddy soil with artificial lightweight material (ALM) manufactured from recycled materials. A total of four treatments, including a control (no amendment), ALM10 (10% of ALM in soil), ALM10+L (10% ALM combined with 0.5% lime), and ALM10+FeO (10% ALM combined with 0.5% FeO), were applied to paddy fields, and rice (*Oryza sativa* L.) was cultivated after 32 weeks. The highest reduction efficiencies for the bioavailable fractions of As and Pb in soil were observed in the ALM10+FeO (52.8%) and ALM10+L treatments (65.7%), respectively. The uptake of As decreased by 52.1% when ALM10+FeO was applied to paddy soil, and that of Pb decreased by 79.7% when ALM10+L was applied. Correlation analysis between bioavailable heavy metals in soil and soil chemical properties showed that soil pH, electrical conductivity (EC), P_2_O_5,_ and soil organic matter (SOM) were the main factors controlling the mobility and bioavailability of As and Pb. Overall, the efficiencies of As and Pb reduction increased synergistically in both soil and plants when FeO and lime were combined with the ALM. In future studies, long-term monitoring is necessary to examine the longevity of soil amendments.

## 1. Introduction

Heavy metal pollution in soil is a critical issue worldwide, particularly in agricultural fields due to food safety concerns [1,2,3,4]. Various sources can contribute to the release of heavy metals into the environment, including industrial areas [5], smelting activities [6,7], and abandoned mines [8,9,10]. Heavy metal pollution in soil or water can be a concern because of its toxicity, persistency, bioaccumulation, and leaching into groundwater [11].

The bioavailable fraction of heavy metals differs from the total heavy metal concentration. The total heavy metal concentration can be used to determine the level of soil contamination, whereas the bioavailable fraction of heavy metals can provide information on the mobility and bioavailability of the heavy metals in soil [12,13,14,15]. Various chemical extractants have been used to estimate the bioavailable fraction of heavy metals in soil. The sequential extraction method is divided into different fractions, such as exchangeable fraction, bound to carbonate fraction, bound to Fe-Mn oxide fraction, bound to organic fraction, and residual fraction depending on ionic strength of the extractants [16]. Among the five different fractions, the exchangeable and bound to carbonate fractions are considered to be the bioavailable fractions of heavy metals [17]. Other extractant includes the toxicity characteristic leaching procedure (TCLP) for conducting ecological risk assessments, Mehlich-3, EDTA, CaCl_2_, and NH_4_NO_3_ [18,19,20,21,22,23].

The application of chemical amendments or adsorbents is a common technique for heavy metal remediation in soil. The main purpose of applying chemical amendments to soil is to immobilize heavy metals, thereby reducing their mobility and bioavailability in soil [24,25,26,27,28,29,30]. Many studies have tried to find amendments or adsorbents that are low cost and have a high remediation efficiency, such as lime, steel slag, coal ash, biochar, and red mud [12,13,31,32,33,34]. Soil amendments have different mechanisms of heavy metal reduction; their reduction efficiencies vary depending on their characteristics and the soil environment. For example, soil pH is one of the factors controlling the mobility and bioavailability of heavy metals. As the soil pH increases, the concentrations of cationic heavy metals in the soil, such as Cd^2+^, Pb^2+^, Pb^2+^, and Cu^2+^ decrease, while the concentration of As increases [12,35,36]. In addition, soil organic matter (SOM) plays an important role in immobilizing the bioavailable fractions of heavy metals. SOM has a high affinity for heavy metals with various functional groups and forms precipitates [31,37,38]. Iron-based materials including iron oxide and zero valent iron have also been used for heavy metal remediation due to their high reactivity, large surface area, and sorption ability [27,35].

Paddy soils provide a unique environment in which anaerobic conditions are maintained during flood periods. Soils with anaerobic conditions have different characteristics than those with aerobic conditions. Prolonged flood conditions in paddy soils can change the microbial activity in soils owing to the anaerobic fermentation of SOM [37]. Under anaerobic conditions, reducing the redox potential can cause the soil pH to become neutral regardless of whether the soil was previously was acidic or alkaline [39]. Consequently, the mobility and bioavailability of heavy metals in paddy soil are different from those of upland soil in terms of the formation of chemical complexes. For example, sulfide, a reduced form of sulfate, can form a metal sulfide complex [40].

Heavy metals including arsenic (As) and lead (Pb) can be accumulated in rice (*Oryza sativa* L.) and cause adverse effects on human health such as cancer, cardiovascular, neurological, and respiratory disease [41]. Heavy metal concentration in paddy soil can be varied depending on the source of pollutants. When groundwater irrigation was the source of as in paddy soil, the concentration of As in soil was ranged 3.1–61.04 mg kg^−1^ in Bangladesh, India, Vietnam, and Nepal [42,43,44]. Mine deposition or industrial wastewater can also be a source of As in paddy soil and concentration of As in soil was ranged 2.5–172.07 mg kg^−1^ in China and 3.05–34.0 mg kg^−1^ in Korea [45,46]. The concentration of Pb in paddy soil was also varied depending on the pollutant source and ranged 24.8–1,486 mg kg^−1^ when mine waste, groundwater, and irrigation surface water were sources of pollutants in paddy soil [34,47,48].

Coal plant by-product such as fly ash and bottom ash has been used for heavy metal remediation in soil because of high porosity and large specific surface area [49]. However, fly ash and bottom ash have a disadvantage in terms of low efficiency for heavy metal remediation in soil when they are used as an original form. For this reason, modification of coal plant by-products such as a hydrothermal method or synthesizing with zeolite adsorbent has been studied to enhance the efficiency of heavy metal remediation [50,51,52,53].

The main purpose of this study was to evaluate the use of recycled waste materials, named artificial lightweight material (ALM), as adsorbents of heavy metal in paddy fields. In addition, ALM combined with FeO and lime was examined for its synergetic effects on the reduction of bioavailable fractions of heavy metals in soil and crops.

## 2. Materials and Methods

### 2.1. Soil Amendments

The main soil amendment, named “Artificial Lightweight Material (ALM),” was provided by a coal power plant located in Incheon province, South Korea. To manufacture ALM, 20% bottom ash, 50% low-quality unburned carbon, and 30% dredging sand were mixed, heated to a temperature of 550–600 °C, cooled to 25 °C, and heated again to 1100–1200 °C in a heating chamber. To evaluate the effects of different combinations of soil amendments, ALM was applied with either 0.5% lime (ALM10+L) or 0.5% iron oxide (ALM10+FeO) in a paddy field. Lime was purchased from a commercially available market, and iron oxide was purchased from Sigma Aldrich.

### 2.2. Determining Application Rate of Soil Amendment

Prior to determining the application rate of ALM in the field, sorption batch experiments were conducted for As and Pb. One hundred mL of each stock solution (100 mg L^−1^), made with NaAsO_2_ and Pb(NO_3_)_2_, was mixed with three different ALM ratios (5%, 10%, and 20% (*w*/*v*)) in a 250 mL flask. The flask was shaken at 120 rpm for 48 h, centrifuged at 3000× *g* for 5 min, and filtered through a 0.45 μm filter paper for ICP-OES analysis. The sorption efficiency of each ALM ratio was determined by calculating the difference between the initial concentration of metals and the remaining concentration in the solution.

### 2.3. Field Experiment Setup

A total of seven, 10 m × 6 m (L × W) plots were constructed in a paddy field and arranged in a completely randomized block design. A 15 cm high guard row was built around each plot to prevent cross-contamination. Each plot was subjected to the following treatments: Control: no amendment; ALM10: ALM 10% (*w/w*); ALM10+L: ALM 10% + 0.5% lime; ALM10+FeO: ALM10% + 0.5% FeO. The plots were thoroughly plowed to make the soil homogeneous and equilibrated for 8 weeks without water supply. The plots were then flooded with water containing trace concentrations of heavy metals (data not shown). A week after flooding, two stands of 50-day old seedling with 3–4 plants in each stand were transplanted, and the flooded condition was maintained for 28 weeks. After 3 weeks of drying, rice was harvested. The rice cultivar named “Oh-Dae”, *Oryza sativa* L., was selected for the experiment as it is a common rice cultivar in the local area. During rice cultivation, conventional cultivation practices, fertilization, and weed control using pesticides were conducted.

### 2.4. Soil and Plant Sampling

Soil samples were collected at 0, 8, 16, and 32 weeks after soil amendment application. Five soil samples were collected from different locations within the treatment area using a hand auger at a depth of 20 cm. These five soil samples were thoroughly mixed to form one composite soil sample, which was placed in a plastic sample bag. The homogenized soil sample was air-dried at room temperature (25 °C) and passed through a 2 mm and then a 0.15 mm sieve for chemical and heavy metal analysis.

Every third stand of rice sample from each treatment plot was collected and air-dried at room temperature (25 °C) until the moisture reached approximately 15%. The rice grains were separated from the plants, and 100 rice grains in each treatment were weighed for growth comparison.

### 2.5. Chemical and Heavy Metal Analysis in Soil and Plant

Soil pH (H_2_O 1:5 *w*/*v*) and electrical conductivity (EC) were measured using a pH meter (MP220, Mettler Toledo, Columbus, OH, USA) and EC meter (S230, Mettler Toledo, Columbus, OH, USA) after shaking 10 g of soil and 50mL of distilled water for 1 h. Soil organic matter (SOM), available P_2_O_5_, and cation exchange capacity (CEC) were measured following the Walkley–Black, Bray No1, and ammonium acetate exchange methods and are summarized in Table 1.

Both As and Pb were extracted by digesting the soil sample with aqua regia (HNO_3_:HCl (*v*/*v*) = 1:3) in a heating block (Block Heating Sample Preparation System, Ctrl-M Science). The bioavailable fractions of the heavy metals in the soil were extracted with the Mehlich-3 extractant (M3).

Digestion of rice grains was conducted in a heating block for 2 h after 24 h of stagnation in an HNO_3_ solution. The heavy metal concentrations were measured using an ICP-OES (ICAP 7000series, Thermo Fisher, Waltham, MA, USA). For quality assurance and quality control purposes, blank and spiked samples were measured every 50 samples. In addition, certified reference material for heavy metal contaminated soil (BAM, Germany) was analyzed, and the mean recovery ratios for As and Pb were 102% and 98%, respectively. All glassware and polyethylene bottles were soaked overnight in a 0.5% HNO_3_ solution and rinsed with deionized water before the experiment. The total content of organic carbon (TOC) and nitrogen (TN) of artificial light material was measured with an elemental analyzer (EA1112, Thermo Fisher Scientific, Waltham, MA, USA).

### 2.6. Statistical Analysis

All measurements were conducted in triplicate, and statistical analysis (ANOVA test) was performed using SPSS software (Version 20.0) [54]. The ANOVA test was conducted using Tukey’s method at a significance level of *p* < 0.05.

## 3. Results and Discussion

### 3.1. Properties of ALM and Paddy Soil

Properties of artificial light material (ALM) are summarized in Table 1. Artificial light material has alkaline properties, a high surface area, and low density. A previous study compared the chemical properties and surface area of ALM to those of bottom ash and showed that ALM had a larger surface area than bottom ash owing to its higher porosity [41]. The main reason for this difference in the morphology of ALM is the hydrothermal treatment of the bottom ash. During the hydrothermal or sintering process of bottom ash, SiO_2_ or Al-containing materials are dissolved, and more porous media can be formed in ALM [55]. In addition, Ca-containing bottom ash is co-precipitated during the sintering process, increasing the alkalinity of ALM [56]. The concentration of As and Pb in ALM was 1.26 and 7.41 mg kg^−1^ and we could expect that application of ALM had minimal effect on heavy metal pollution in paddy soil.

The initial chemical properties of the paddy fields are shown in Table 2. Soil pH was close to neutral. The EC (<2.0 dS m^−1^), SOM (2.0–4.0%), P_2_O_5_ (80–120 mg kg^−1^), and CEC (10–15 cmol kg^−1^) were in the optimum range for crop growth set by the Rural Development Agency in Korea. However, the total heavy metal concentration in paddy fields exceeded the threshold value set by the Ministry of Environment in Korea, except for that of Cu. In particular, the concentration of As was approximately 14 times higher than the threshold value, indicating that the study area was highly polluted with As.

### 3.2. Determining Optimum Application Rate of Amendments

**Figure 1:**
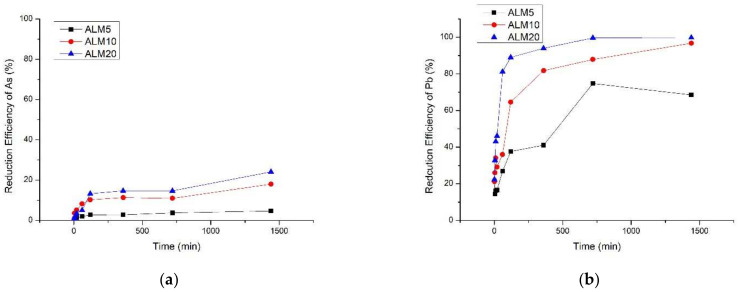
Reduction efficiency of (**a**) As and (**b**) Pb in aqueous solution for determining optimum application rate of amendments.

An aqueous batch experiment was conducted to determine the optimum application rate of soil amendments. Three different ratios (5, 10, and 20% *w*/*v*) of ALM were added to a solution containing 100 mg L^−1^ of As and Pb. The sorption efficiency was calculated by measuring the heavy metal concentration in the solution at each time interval for 24 h (Figure 1).

For both As and Pb, the sorption efficiency increased as the amount of added ALM increased. The highest sorption efficiency was observed when 20% of ALM was added to the solution (24.1% for As and 99.9% for Pb). However, the optimum application rate of ALM in soil was determined to be 10% because of the high pH of ALM. In addition, the sorption efficiencies for As and Pb were 17.9% and 96.8%, respectively, when 10% of ALM was applied in the solution, indicating that there was no significant difference in sorption efficiency between the 10% and 20% application of ALM.

### 3.3. Changes of Chemical Properties in Paddy Soil after Application of Amendments

Soil pH, EC, SOM, P_2_O_5,_ and CEC measured immediately after harvesting (32 weeks after sowing) are summarized in Table 3. Soil pH in all treatments, including the control, was close to neutral, and a slight decrease in soil pH was observed after applying the amendments. Application of ALM 10% had no significant effect on the chemical properties of the soil except available P_2_O_5_ decreasing by 29.9% and the addition of lime increased the CEC of the soil by 23.5% compared to the control. In the case of FeO addition, the SOM and CEC decreased by 23.8% and 30.2%, respectively, compared to the control. Decreasing availability of P_2_O_5_ in the ALM10 application could be contributed by the high porosity of ALM and mixing lime and FeO with ALM may cause decreasing sorption site for phosphorus.

### 3.4. Effect of Amendments on Bioavailability of Heavy Metals in Soil

The total heavy metal concentration has limited utility in evaluating the mobility or bioavailability of heavy metals in soil because of matrix complexity [37,57]. For this reason, various extractants are used to estimate the bioavailability of heavy metals in soil. Mehlich-3 (M3) extractant was used in this study. The M3 extractant has been commonly used to estimate the bioavailability and mobility of heavy metals in soil and plants [15,58,59]. The concentrations of As and Pb in the soil extracted with M3 are summarized in Table 4. After the application of the amendments, the bioavailable fractions of heavy metals significantly decreased compared to those of the control. When ALM10 was applied to the soil in paddy fields, the reduction efficiencies for the bioavailable fractions were 22.7% for As and 52.4% for Pb. However, the highest reduction efficiencies for As (52.8%) and Pb (65.7%) were observed when ALM10 was combined with FeO and lime.

A previous study revealed that the main heavy metal removal mechanism of ALM in an aqueous solution was either chemical precipitation or complexation with oxygen and sulfur [32]. In the case of Cd and Pb, ALM formed complexes using its –OH, -CO_3_, -O, and –S functional groups, and Cd(OH)_2_, CdCO_3_, PbS, and PbO were formed at the surface of ALM. When ALM was combined with FeO, a synergetic reduction effect was observed for As in the soil. Aftabtalab et al. (2022) outlined that the bioavailable fraction of As in soil is highly related to dissolved organic matter (DOM) and FeO concentrations. The As in soil is typically removed by sorption onto the surface of metal oxides, with FeO being one of the main chemical species present [35]. In addition, DOM strongly interacts with FeO and consequently affects the mobility and bioavailability of As in soil. A previous study also revealed that As sorption onto iron oxide (α-γ-Fe_2_O_3_), crystalline iron oxide, and various other forms of iron oxide occur in the presence of DOM [60,61].

In the case of lime addition combined with ALM, increased soil pH is one of the main reasons for the reduction in the concentration of bioavailable Pb. The addition of lime as an amendment increases soil pH. This increases the negative charges on the surface of the soil; consequently, the sorption efficiencies of cationic heavy metals such as Pb, and Cd are increased [13,62]. In addition, under anaerobic conditions during flood periods in paddy soils, SO_4_^2−^ can contribute to the removal of Pb from the soil. Cao et al. (2011) and Wang et al. (2015) observed that SO_4_^2−^ can form complexes with Pb^2+^ in soil solution and form precipitates such as Pb_2_(SO_4_)O and Pb_4_(CO_3_)_2_(SO_4_)(OH)_2_ [31,33]. Although our study did not measure the SO_4_^2−^ concentrations in soil, we may assume that SO_4_^2−^ contributed to the removal of Pb from anaerobic conditions of paddy soil.

### 3.5. Heavy Metal Accumulation in Rice

The concentrations of accumulated As and Pb in rice subjected to different treatments are summarized in Table 5. A significantly lower concentration of As compared to the control was observed when ALM10 and ALM10+FeO were applied to the soil. The highest reduction efficiency was observed with the ALM10+FeO treatment (52.1%), followed by the ALM10 treatment (31.3%). All three treatments reduced the bioavailable fraction of Pb in rice. The highest reduction efficiency was observed with the ALM10+L treatment (79.7%), followed by the ALM10+FeO treatment (71.5%) and the ALM10 treatment (62.0%). Comparing the reduced concentrations of As and Pb in rice, a higher reduction efficiency of Pb was observed than that of As in all three treatments. The reduction efficiency of bioavailable As and Pb ranged from 13.0–52.1% to 62.0–79.7%, respectively.

The bioaccumulation mechanisms of As and Pb are different [4,63]. Bioaccumulation of As in rice mainly occurs via the phosphate transporter for As(V) and nodulin 26 type-intrinsic proteins for As (III), along with plasma membrane intrinsic proteins (PIP) or tonoplast intrinsic proteins (TIP). In anaerobic conditions, such as those in the subsurface of paddy soil, microbial respiration can convert inorganic As to methylated-As. In this case, aquaporin channels used as silicate transporters are the main translocation pathways for methylated-As in rice [2]. In the case of Pb, most of the absorbed bioavailable Pb is accumulated at the surface of root cells in the form of iron plaque [34]. Iron plaque has a high functional group sorption capacity and promotes the immobilization of Pb [64]. In addition, the main ionic form of Pb, Pb^2+^, forms immobilized precipitates such as 2PbCO_3_·Pb(OH)_2_ and Fe-ferri-hydrite in the root. This inhibits the translocation of Pb from root to grain [47].

After harvesting the rice, the weight of 100 grains was measured (data is in Supplementary Table S1). Compared to the control (3.03 g), the weight of rice grain was reduced 1.9–6.6% when the amendment was applied in the plot. However, no significant difference was observed for rice grain weight in each plot.

### 3.6. Correlation Analysis between Chemical Properties and Heavy Metal Concentration in Soil

The bioavailable fractions of As and Pb extracted with M3 in soil were analyzed to examine their correlation with soil chemical properties, the results are summarized in Table 6. Soil chemical properties can greatly impact the uptake of heavy metals from soil to crop systems [65]. Soil pH, EC, P_2_O_5,_ and SOM were significantly correlated with the bioavailable fraction of As. This result agrees with that of Zhou et al. (2014), who found that soil pH and CEC are the main factors controlling the immobilization of bioavailable heavy metals in soil [57]. The cationic forms of heavy metals such as Pb^2+^, Cd^2+^, and Pb^2+^ were reduced when soil pH and CEC were increased. Our results also showed a significant negative correlation between Pb and soil pH (*p* < 0.01). Although no significant difference was observed between CEC and Pb concentration in soil, a negative correlation indicates that when CEC was higher, a lower bioavailable fraction of Pb was measured. Therefore, we concluded that soil pH was the main factor contributing to the reduction of the bioavailable fraction of Pb in this study through surface complexation reaction of the cationic form of heavy metals and possibly, ionic exchange or metal-binding exchange mechanisms [41,66].

In the case of the bioavailable fraction of As, the opposite correlation to Pb was observed, except for SOM and P_2_O_5_. A significant positive correlation was observed between As and soil pH and, EC. CEC also showed a slight positive correlation with As, but it was insignificant. However, a significantly negative correlation was observed between SOM and the bioavailable fraction of As in soil. This result indicates that as soil pH, EC, and CEC concentration increased, the bioavailable fraction of As in the soil also increased. In addition, higher SOM and P_2_O_5_ led to lower As concentrations in the soil. This result agrees with a previous study showing that a high SOM concentration and low soil pH can contribute to reducing the bioavailable fraction of As in paddy soil [1,2,35,38,67]. In the case of P_2_O_5_, phosphorus has a similar chemical behavior and uptake pattern. Furthermore, As and P_2_O_5_ can be competitive for sorption sites on sorbent [68]. Those properties might impact a negative correlation between bioavailable As concentration and P_2_O_5_ concentration in soil.

## 4. Conclusions

ALM produced from recycled waste, bottom ash, unburned coal, and dredged sand was examined for heavy metal remediation in soil. The application of ALM combined with FeO and lime was examined to determine their synergetic effects in reducing the bioavailable fraction of heavy metals. When ALM was applied to paddy fields, the bioavailable fractions of As and Pb decreased by 22.7% and 52.4%, respectively. However, when ALM was combined with FeO and lime, the reduction efficiencies of As and Pb significantly increased by 52.8% and 65.7%, respectively, compared to those in the control. In addition, the uptake of As and Pb also decreased by 52.1% and 79.7%, respectively, when ALM was combined with FeO and lime.

Correlation analysis showed that soil pH, EC, P_2_O_5_, and SOM were the main factors that reduced the bioavailable fractions of heavy metals, and opposing correlations were observed for As and Pb. When the soil pH and EC increased, the reduction efficiency of As decreased, whereas the reduction efficiency of Pb increased as the soil pH and EC decreased. In the case of SOM, As and Pb concentrations decreased when SOM concentration was increased.

Although the main reduction mechanism was not confirmed, the increases in soil pH, EC, and CEC concentrations may affect to reduce the heavy metal concentrations in the soil through ionic exchange and chemical complexation. In addition, combining FeO and lime with ALM showed a synergetic effect in reducing the bioavailable fractions of As and Pb in soil. However, we have not examined the longevity of applied amendments in the field and in the future, long-term monitoring of the mobility and bioavailability of heavy metals should be conducted to assess the longevity of amendments in soil.

## Figures and Tables

**Table 1:** Physicochemical properties of artificial light material (ALM) used in this experiment.

			ALM
pH		(H_2_O, 1:5 *w*/*v*)	8.68 ± 0.11
EC		dS m^−1^	1.29 ± 0.03
TOC ^†^		g kg^−1^	45.4 ± 0.4
TN ^†^		g kg^−1^	0.5 ± 0.1
Surface area		m^2^ g^−1^	7.8477
Density		g cm^−3^	0.82
Heavy metals	As	mg kg^−1^	1.26 ± 0.61
	Cd	mg kg^−1^	0.22 ± 0.02
	Pb	mg kg^−1^	7.41 ± 1.37
	Cu	mg kg^−1^	5.13 ± 0.05
	Zn	mg kg^−1^	14.52 ± 4.49

^†^ TOC and TN are the abbreviations of total organic carbon and total nitrogen.

**Table 2:** The initial physicochemical properties of paddy soil before the experiment.

			Soil	Optimum Range/Threshold Value ^†^
pH		(H_2_O, 1:5 *w*/*v*)	7.30 ± 0.02	6.0–7.0
EC ^§^		dS m^−1^	1.82 ± 0.02	<2.0
SOM ^§^		%	3.12 ± 0.01	2.5–3.0
P_2_O_5_		mg kg^−1^	83.0 ± 12.5	80–120
CEC ^§^		cmol kg^−1^	14.7 ± 0.23	10–15
Heavy metals	As	mg kg^−1^	350.9 ± 11.2	25
	Cd	mg kg^−1^	5.33 ± 0.95	4
	Pb	mg kg^−1^	207.5 ± 15.3	200
	Cu	mg kg^−1^	33.54 ± 5.32	150
	Zn	mg kg^−1^	168.9 ± 26.4	300

^†^ Optimum range for soil chemical properties and threshold value for heavy metal concentration. ^§^ EC, SOM, and CEC are the abbreviation for electric conductivity, soil organic matter, and cationic exchange capacity.

**Table 3:** Soil chemical properties in paddy field after applying amendment.

Treatment	pH	EC	SOM	P_2_O_5_	CEC
		dS m^−1^	%	mg kg^−1^	cmol kg^−1^
Control	7.29 ± 0.06 ^a^	0.88 ± 0.05 ^b^	4.15 ± 0.12 ^a^	78.2 ± 13.2 ^a^	14.62 ± 0.22 ^b^
ALM10	6.93 ± 0.03 ^a^	1.03 ± 0.11 ^b^	3.68 ± 0.08 ^ab^	54.8 ± 19.4 ^b^	13.09 ± 0.18 ^b^
ALM10+L	7.11 ± 0.05 ^a^	1.28 ± 0.16 ^a^	4.08 ± 0.15 ^a^	62.4 ± 8.9 ^ab^	19.12 ± 0.15 ^a^
ALM10+FeO	7.12 ± 0.02 ^a^	0.89 ± 0.06 ^b^	3.16 ± 0.04 ^b^	61.2 ± 17.8 ^ab^	10.20 ± 0.17 ^c^

Different letters indicate that the value is significantly different at *p* < 0.05.

**Table 4:** Bioavailable fraction of As and Pb in soil after application of amendments.

Treatments	As	Pb
	mg kg^−1^	mg kg^−1^
Control	1.63 ± 0.03 ^a^	12.99 ± 0.36 ^a^
ALM10	1.26 ± 0.06 ^b^	6.18 ± 0.26 ^c^
ALM10+L	1.51 ± 0.07 ^ab^	4.46 ± 0.15 ^d^
ALM10+FeO	0.77 ± 0.04 ^c^	10.33 ± 0.32 ^b^

Different letters indicate that value is significantly different at *p* < 0.05.

**Table 5:** Concentration of As and Pb in rice grain harvested after the experiment.

Treatments	As	Pb
	mg kg^−1^	mg kg^−1^
Control	1.92 ± 0.33 ^a^	1.58 ± 0.18 ^a^
ALM10	1.32 ± 0.17 ^c^	0.60 ± 0.04 ^b^
ALM10+L	1.67 ± 0.21 ^b^	0.32 ± 0.07 ^c^
ALM10+FeO	0.92 ± 0.14 ^d^	0.45 ± 0.09 ^bc^

Different letters indicate that value is significantly different at *p* < 0.05.

**Table 6:** Correlation analysis between bioavailable heavy metals and soil chemical properties.

	As	Pb
pH	0.913 ** (0.001)	−0.882 ** (0.001)
EC	0.605 * (0.037)	−0.723 ** (0.008)
SOM	−0.848 ** (0.001)	−0.575 (0.051)
P_2_O_5_	−0.871 ** (0.004)	−0.251 (0.061)
CEC	0.267 (0.401)	−0.031 (0.924)
As	1.000	−0.693 * (0.012)
Pb		1.000

Pearson correlation coefficient (*p*-value) is shown. * and ** represent that *p* value is significant at *p* < 0.05 and *p* < 0.01, respectively.

## Data Availability

The data presented in this study are available on request from the corresponding author.

## Supplementary Materials

The following supporting information can be downloaded at https://www.mdpi.com/article/10.3390/toxics10020090/s1, Table S1: Weight of 100 rice grain measured after harvesting.
