# Supplementary material for: Effect of Combined Soil Amendment on Immobilization of Bioavailable As and Pb in Paddy Soil
Source: Toxics. 2022 Feb 16;10(2):90. doi: 10.3390/toxics10020090 (PMC8878171; doi:10.3390/toxics10020090)
Supplement: Supplementary file 1 [file toxics-10-00090-s001.zip › toxics-1570558-supplementary.pdf]

toxics

MDPI

Supplementary Materials: Effect of Combined Soil Amendment on Immobilization of Bioavailable As and Pb in Paddy Soil

Young-Kyu Hong, Jin-Wook Kim, Sang-Phil Lee, Jae-E. Yang and Sung-Chul Kim

Table S1. Weight of 100 rice grain measured after harvesting.

	Grain weight
	g
Control	3.03 ± 0.01 [a]
ALM10	2.83 ± 0.04 [a]
ALM10+L	3.03 ± 0.12 [a]
ALM10+FeO	2.97 ± 0.02 [a]

[a] Significance was evaluated at $p < 0.05$.
